# Probing pairing symmetry in multi-band superconductors by quasiparticle interference

**DOI:** 10.1038/s41598-018-30045-0

**Published:** 2018-08-02

**Authors:** A. Dutt, A. A. Golubov, D. V. Efremov, O. V. Dolgov

**Affiliations:** 1 Faculty of Science and Technology and MESA+ Institute of Nanotechnology, University of Twente, 7500 AE Enschede, The Netherlands; 2 Moscow Institute of Physics and Technology, Dolgoprudnyi, Moscow region, Russia; 3 Leibniz-Institut für Festkörper- und Werkstoffforschung Dresden, Dresden, 01069 Germany; 4 Max-Planck-Institut FKF, D-70569 Stuttgart, Germany; 5 P.N. Lebedev Physical Institute, RAS, Moscow, Russia

## Abstract

We study momentum and energy dependencies of the quasiparticle interference (QPI) response function in multiband superconductors in the framework of the strong-coupling Eliashberg approach. Within an effective two-band model we study the *s*_±_ and *s*_++_ symmetry cases, corresponding to opposite or equal signs of the order parameters in the bands. We demonstrate that the momentum dependence of the QPI function is strikingly different for *s*_±_ and *s*_++_ symmetries of the order parameter at energies close to the small gap. At the same time, the QPI response becomes indistinguishable for both symmetries at higher energies around the large gap. This result may guide future experiments on probing pairing symmetry in iron pnictides as well as in other unconventional superconductors.

## Introduction

Recent developments of the tunneling spectroscopy technique have allowed to make a great progress in elucidating the physics of high-temperature superconductors^1–4^. In particular, the analysis of quasiparticle interference provided information about the phase shift of the superconducting order parameter between different bands in most of the Fe-based superconductors (FeBS), which are in focus of research during past years^5–8^.

Most of the physical properties of FeBS are well established, but the symmetry of the superconducting order parameter is still under discussion. The studies of the thermodynamics of FeBS, and also the ARPES, revealed that the superconducting order parameter is a singlet and has a nodeless s-wave symmetry. Further investigations of the inelastic neutron and QPI spectra allowed to determine that the optimally doped FeBS have the superconducting order parameter with different signs on the electron and hole Fermi pockets, the so-called and *s*_+−_ symmetry state. At the same time, there are materials with only electron bands, as in AFe_2_Se_2_, or hole bands, as in AFe_2_As_2_ (here A = K, Rb, Cs), in which the symmetry of the superconducting order parameter is different from *s*_+−_. Moreover, some of the recent experiments and theoretical investigations show that the sign can be changed either with doping or by an increase of disorder^9–17^. Therefore the important question to be addressed is, how universal is *s*_±_ symmetry.

In the previous works^18,19^, it has been established that the qualitative determination of the superconducting order parameter is possible by studying the energy dependence of QPI spectra within the frequency window close to the small band gap. In the present paper, we explore the momentum dependence of the QPI-response function *I*(**q**, *ω*) to show the relation of the developed theory to experimental works, which use the momentum dependent QPI-response function at fixed energies. In the framework of the two-band Eliashberg model we show that *Q*(**q**, *ω*) is strikingly different for *s*_++_ and *s*_±_ symmetries of the superconducting order parameter at energies close to the small gap.

The paper is organized as follows: In Sec. 2 we describe the model of QPI in the low energy limit. Under Sec. 3 we present the results for the momentum dependence of QPI response function in a two-band model, consisting of an electron and a hole band. The behavior by which the QPI response function deviates from the conditions of perfect nesting, i.e. its dependence upon the ellipticity of the electron bands and the energy mismatch *δμ* between the two band pockets were explored. In the Sec. 4 the possible case for scattering between two electron (hole) pockets is discussed. Finally, in Sec. 5 we give the summary and the conclusion made from the data analysis.

## The model

In general, the single-particle correlation functions, including *I*(***q***, *ω*), in multiband systems with strong coupling interaction can be found by using the multiband extension of the Eliashberg theory^20–27^. The theory can be applied to Fe-based superconductors, since the Fermi surface of the moderately doped compounds consists of two or three relatively small and almost circular, hole-like pockets at Γ = (0, 0) and two elliptic electron pockets at M = (*π*, 0) and (0, *π*) points^28–31^. Furthermore, due to the small anisotropy of the order parameter in Fe-based superconductors, the good description can be achieved with use of *ξ*-integrated quasiclassical Green functions ${\hat{{\bf{g}}}}_{\alpha }(\omega )={N}_{\alpha }(0)\int d\xi {\hat{{\bf{G}}}}_{\alpha }(k,\omega )$, where *α* is the band index. Further simplification can be done by consideration of an effective two-band model, since it was shown in^32^ that the problem in the clean limit can be treated in such representation. In the Nambu notations the full Green functions have the form:

1 $${\hat{{\bf{G}}}}_{\alpha }({\bf{k}},\omega )=\frac{{\tilde{\omega }}_{\alpha }{\hat{\tau }}_{0}+{\xi }_{\alpha ,{\bf{k}}}{\hat{\tau }}_{3}+{\tilde{\varphi }}_{\alpha }{\hat{\tau }}_{1}}{{\tilde{\omega }}_{\alpha }^{2}-{\xi }_{\alpha ,{\bf{k}}}^{2}-{\tilde{\varphi }}_{\alpha }^{2}},$$

where the ${\hat{\tau }}_{i}$ denote Pauli matrices in Nambu space. Here *ξ*_*α*,***k***_ = *ε*_*α*,***k***_ − *ε*_*F*_ is the linearized dispersion at the Fermi energy. The order parameter ${\tilde{\varphi }}_{\alpha }$ and the renormalized frequency ${\tilde{\omega }}_{\alpha }$ are complex functions of the frequency *ω*. Through the text we use retarded Green functions omitting the index R. The quasiclassical *ξ*-integrated Green functions ${\hat{{\bf{g}}}}_{\alpha }(\omega )={g}_{0\alpha }{\hat{\tau }}_{0}+{g}_{1\alpha }{\hat{\tau }}_{1}$ are obtained by numerical solution of the Eliashberg equations^21^

2 $${\tilde{\omega }}_{\alpha }(\omega )-\omega =\sum _{\beta }{\int }_{-\infty }^{\infty }dz{K}_{\alpha \beta }^{\tilde{\omega }}\,(z,\omega )Re\frac{{\tilde{\omega }}_{\beta }(z)}{\sqrt{{\tilde{\omega }}_{\beta }^{2}(z)-{\tilde{\varphi }}_{\beta }^{2}(z)}},$$

3 $${\tilde{\varphi }}_{\alpha }(\omega )=\sum _{\beta }{\int }_{-\infty }^{\infty }dz{K}_{\alpha \beta }^{\tilde{\varphi }}(z,\omega )Re\frac{{\tilde{\varphi }}_{\beta }(z)}{\sqrt{{\tilde{\omega }}_{\beta }^{2}(z)-{\tilde{\varphi }}_{\beta }^{2}(z)}}.$$

The kernels ${K}_{\alpha \beta }^{\tilde{\varphi },\tilde{\omega }}(z,\omega )$ of the fermion-boson interaction have the standard form ?:

4 $${K}_{\alpha \beta }^{\tilde{\varphi },\tilde{\omega }}(z,\omega )={\int }_{-\infty }^{\infty }\,\,d{\rm{\Omega }}\frac{{\lambda }_{\alpha \beta }^{\tilde{\varphi },\tilde{\omega }}B({\rm{\Omega }})}{2}[\frac{\tanh \,\frac{z}{2T}+\,\coth \,\frac{{\rm{\Omega }}}{2T}}{z+{\rm{\Omega }}-\omega -i\delta }].$$

**Figure 1:**
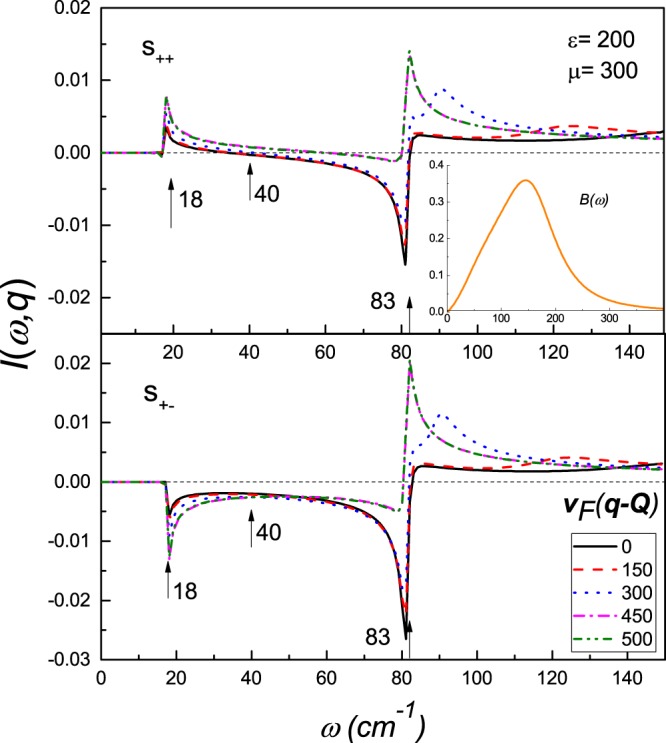
The QPI response function I(*ω*, **q**) of the scattering between the electron and the hole bands, for the *s*_++_ and *s*_±_ symmetry of the superconducting order parameter at various momentum values with parameters as, *ε* = 200, *δμ* = 300 and *ϕ* = 0 with *β* = −1 at *T* = 1 *cm*^−1^. The transition temperature is *T*_*c*_ = 28 *cm*^−1^. Inset in the upper panel: the spectral function of the electron-bosonic interaction *B*(Ω).

For simplicity, we use the same normalized spectral function of the electron-boson interaction *B*(Ω) obtained for spin fluctuations in inelastic neutron scattering experiments^33^ for all the channels. The maximum of the spectra is Ω_*sf*_ = 144 *cm*^−1^ (Fig. 1), which determines the natural energy scale^34,35^. This spectrum gives a rather good description of the thermodynamical^32^ and optical^36,37^properties in the SC as well as normal states^38^. The matrix elements ${\lambda }_{\alpha \beta }^{\tilde{\varphi }}$ are positive for attractive interactions and negative for repulsive ones. Further for simplicity we will omit the subscripts $\tilde{\omega }$ and $\tilde{\varphi }$ denoting ${\lambda }_{\alpha \beta }^{\tilde{\varphi }}={\lambda }_{\alpha \beta }$ and ${\lambda }_{\alpha \beta }^{\tilde{\omega }}=|{\lambda }_{\alpha \beta }|$.

Scanning tunneling spectroscopy provides the differential conductance which is proportional to the local single particle density of states *N*(*r*, *ω*)

$$\frac{dI}{dV}({\bf{r}},\omega )\propto |g({\bf{r}}){|}^{2}N({\bf{r}},\omega ),$$

where *g*(***r***) is the local tunneling matrix element. The local density of states is related to the single particle retarded Green functions:

5 $$N(r,\omega )=-\,\frac{1}{\pi }{\rm{Im}}Tr\,[\frac{1+{\tau }_{3}}{2}\hat{G}({\bf{r}},{\bf{r}},\omega )]$$

Here *Tr*[..] is taken over both Nambu and band indices. In the linear response approximation the perturbation of the density of states due to an impurity with the point-like scattering $\hat{U}(r)={U}_{\alpha \beta }\delta (r){\tau }_{3}$ reads:

6 $$\delta N({\bf{r}},\omega )=-\,\frac{1}{\pi }Im\sum _{\alpha ,\beta }Tr\,[\frac{1+{\tau }_{3}}{2}\int dV^{\prime\prime} {\hat{G}}_{clean}^{\alpha }({\bf{r}}-{\bf{r}}^{\prime\prime} ,\omega ){\hat{U}}_{\alpha \beta }({\bf{r}}^{\prime\prime} ){\hat{G}}_{clean}^{\beta }({\bf{r}}^{\prime\prime} -{\bf{r}},\omega )]$$

where ${\hat{G}}_{clean}^{\beta }({\bf{p}},\omega )$ is the Green function in the clean case.

Below we consider the Fourier transform of *δN*(**r**, *ω*), according to the usual experimental procedure:

$$\delta N({\bf{r}},\omega )=\sum _{\alpha \beta }{U}_{\alpha \beta }\int \frac{{d}^{2}q}{{\mathrm{(2}\pi )}^{2}}{e}^{i{\bf{qr}}}I({\bf{q}},\omega ),$$

where the response function reads:

7 $$I({\bf{q}},\omega )=-\,\frac{1}{2\pi }\sum _{\alpha \beta }\int \frac{{d}^{2}p}{{(2\pi )}^{2}}ImTr[{\tau }_{3}{\hat{G}}_{clean}^{\alpha }({\bf{q}}+{\bf{p}},\omega ){\tau }_{3}{\hat{G}}_{clean}^{\beta }({\bf{p}},\omega )].$$

In this approximation, the QPI response function is considered as a sum over response functions of the quasiparticle scattering between pairs of the pockets. Therefore the problem can reduced to an effective two-pocket model. To illustrate the most essential effects, below we will use the Fermi surface containing two pockets (see, Fig. 1), one hole-like and one electron-like. Correspondingly we linearize the energy spectrum near the Fermi-level:

$$\begin{array}{rcl}{\xi }_{{\rm{\Gamma }}}({\bf{p}}) & = & {v}_{{F}_{{\rm{\Gamma }}}}({\bf{p}}-{{\bf{p}}}_{{F}_{{\rm{\Gamma }}}})\\ {\xi }_{M}({\bf{p}}+{\bf{Q}}) & = & {v}_{{F}_{{\bf{M}}}}({{\bf{p}}}_{{F}_{{\bf{M}}}}(\theta )-{\bf{p}})\\  &  & \sim {v}_{{F}_{{\bf{M}}}}({{\bf{p}}}_{{F}_{{\bf{M}}}}-{\bf{p}})+\varepsilon \,\cos \,2\theta .\end{array}$$

**Figure 2:**
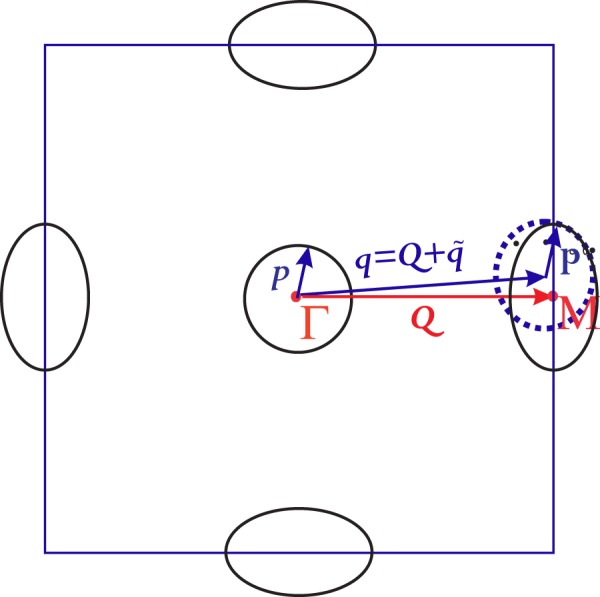
Schematic plot of the band structure. The blue dashed circle represents the shift of the hole pocket at Γ by ${\bf{q}}={\bf{Q}}+\tilde{{\bf{q}}}$.

Here **Q** is the vector that connects the centres of the pockets situated at Γ and the ***M*** points, respectively, *ε* characterizes the ellipticity of the electron bands (as shown in Fig. 2) and *θ* is the angle between **p** and *x* -axis. This expansion is valid for the momenta ***p*** that lie close to the Fermi surface and the ellipticity parameter obeys the condition that $|\varepsilon |\ll {{\bf{v}}}_{F}{{\bf{p}}}_{F}$. To keep the generality we will use the notation for the pockets as *a* and *b*. It is pertinent to introduce the chemical potential difference between the two bands such as *δμ* = **v**_*Fb*_(**p**_*Fa*_ − **p**_*Fb*_). Next, we introduce the parameter *β* = *v*_*Fb*_/*v*_*Fa*_ for the electron-electron and hole-hole band scattering and *β* = −*v*_*Fb*_/*v*_*Fa*_ for electron-hole band scattering. The above approximation yields

8 $$\begin{array}{rcl}{\xi }_{b}({\bf{p}}+{\bf{q}}) & \approx  & \beta {\xi }_{a}({\bf{p}})+{v}_{Fb}\tilde{q}\,cos(\theta -\varphi )\\  &  & +\varepsilon \,\cos \,2\theta +\delta \mu .\end{array}$$

Here, ${\bf{q}}={\bf{Q}}+\tilde{{\bf{q}}}$ and *ϕ* is the angle between momentum vectors $\tilde{{\bf{q}}}$ and **Q**. The general expression for the response function, considering a constant density of states, has the following form^18^

9 $$I(\omega ,{\bf{q}})=-\,\frac{\sqrt{{N}_{a}{N}_{b}}}{2}Im[K(\omega )F(\omega ,{\bf{q}})],$$

where *K*(*ω*) is the coherence factor as follows

10 $$K(\omega )=[\frac{{\tilde{{\rm{\Delta }}}}_{a}{\tilde{{\rm{\Delta }}}}_{b}-{\omega }^{2}}{{E}_{a}{E}_{b}}+{\rm{sgn}}(\beta )].$$

Here ${\tilde{{\rm{\Delta }}}}_{\alpha }(\omega )={\tilde{\varphi }}_{\alpha }(\omega )/{Z}_{\alpha }(\omega )$ and ${Z}_{\alpha }(\omega )={\tilde{\omega }}_{\alpha }(\omega )/\omega$ and are complex functions. Here, ${E}_{\alpha }(\omega )=\sqrt{{\omega }^{2}-{\tilde{{\rm{\Delta }}}}_{\alpha }^{2}(\omega )}$ is the quasiparticle energy spectrum. To find the single particle gap function ${\tilde{{\rm{\Delta }}}}_{\alpha }(\omega )$ and the renormalization function *Z*(*ω*), we employ the Eliashberg approach^21,27^. The angle averaged *F*-function in Eq. (9) reads:

11 $$F(\omega ,{\bf{q}},\varphi )={\langle \frac{{\mathscr{Z}}(\omega )}{{({\mathscr{Z}}(\omega ))}^{2}+|\beta |{({\mathscr{Y}}({\bf{q}},\varphi ,\theta ))}^{2}}\rangle }_{\theta }$$

where we define ${\mathscr{Z}}(\omega )=\sqrt{|\beta {|}^{-1}}{Z}_{a}{E}_{a}+\sqrt{|\beta |}{Z}_{b}{E}_{b}$ and ${\mathscr{Y}}({\bf{q}},\varphi ,\theta )=\varepsilon \,\cos (2\theta )+\delta \mu +{v}_{b}\tilde{q}\,\cos (\theta -\varphi )$. In this paper, we are focussing completely on the inter-band scattering (at the vector ${\bf{q}}={\bf{Q}}+\tilde{{\bf{q}}}$) aspect of the phenomenon and parameter dependence of the response function at temperature *T* = 1, near the smaller band gap energy Δ_*b*_.

## Results and Discussion

In this section, we study the evolution of the momentum and the energy dependence of the QPI response function *I*(*ω*, **q**), with the change of the band parameters *viz*., the ellipticity of electron-like bands *ε*, the Fermi energy mismatch *δμ* (only finite and large value) between the bands and momentum parameter ${{\bf{v}}}_{b}\tilde{{\bf{q}}}$. The model under consideration is schematically shown in Fig. 2.

For the calculation of the superconducting gap function we use the Eliashberg procedure with the spin-fluctuation spectral function^9^ having a peak frequency of Ω_*sf*_ = 144 *cm*^−1^. The energy gaps value for the largest gap is Δ_*a*_(*ω*) = 83 *cm*^−1^ and the smallest gap is Δ_*b*_(*ω*) = 18 *cm*^−1^ at zero temperature. We will use *cm*^−1^ as the units of energy throughout the text. The coupling *λ* -matrix has the elements defined as *λ*_*aa*_ = 3, *λ*_*ab*_ = ±0.2, *λ*_*ba*_ = ±0.1, *λ*_*bb*_ = 0.5, with “+” sign for the *s*_++_ and “−” sign for the *s*_±_ symmetry case, for data obtained in all the figures.

In Fig. 2, we plot *I*(*ω*, **q**) as a function of *ω* at various momenta for the *s*_++_ and *s*_±_ symmetry, respectively. The QPI response function for fixed ***q*** has two or three extrema. The first two are strong peaks/dips, which are located at the energy of the superconducting gaps Δ_*a*_ and Δ_*b*_. The third one is rather weak, has a strong **q** dependence and resides at the energy larger than Δ_*a*_. The last one has the same character for both gap symmetries, and therefore, cannot serve as an indicator of the symmetry of the superconducting order parameter.

Comparing the first two extrema for *s*_++_ and *s*_±_ symmetry of the superconducting order parameters, one can make an interesting observation. While *I*(*ω*, **q**), at the energy of the largest gap, shows a maximum for both symmetries of the order parameter, at the energy of the smallest gap, it has a different character, i.e. for *s*_++_ it has a maximum, and for *s*_±_, a minimum. It makes the energy windows close to the smallest gap, an important tool for determination of the symmetry of the superconducting gap^18^. Further, the examination of the evolution of QPI function with momentum, shows that, it has non-monotonic character and demands a special consideration.

**Figure 3:**
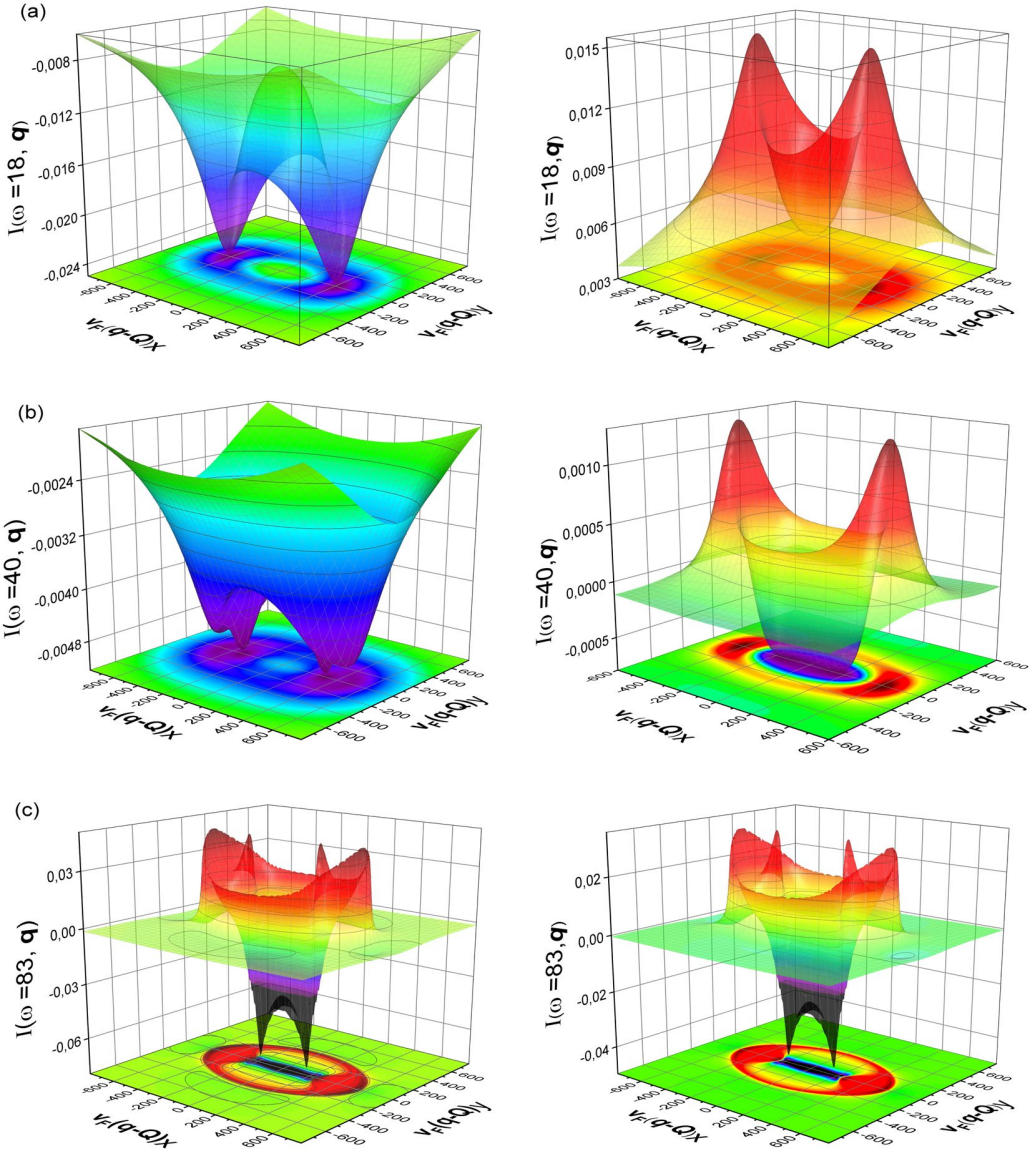
Momenta dependence of the QPI response function for the scattering between an electron and a hole band, with parameters chosen as, *δμ* = 300 and *ε* = 200,and *β* = −1 at certain energies (i.e. ω = Δ_*b*_, ${{\rm{\Delta }}}_{b} < \omega  < \,{{\rm{\Delta }}}_{a}$ and ω = Δ_*a*_). Here, the panels (**a**),(**b**),(**c**) correspond to the *s*_±_ (left) and *s*_++_ (right), respectively.

In Fig. 3, we show the 3D plots depicting the momentum dependence of *I*(*ω*, **q**) taken at the energy of the small band gap, at the energy of the large gap and at the energy between the large and the small gaps. The momentum dependence of the QPI response function at the energy close to Δ_*b*_ is depicted in Fig. 3(a). The response function is positive for all momenta in the case of *s*_++_ symmetry and negative for all momenta for the *s*_±_ symmetry. It confirms the conclusion of the work^18^ of the possibility to use the QPI response function at the energy of the small gap as an indicator of the symmetry of the superconducting order parameter. Further analysis of the momentum dependence of the QPI response function shows that the peaks in *I*(*ω* = Δ_*b*_, **q**) correspond to certain momenta, i.e. the case, when shifted by vector **q**, the pocket at Γ point touches the Fermi surface pocket at *M* point, as is show in Fig. 1.

For intermediate energy at *ω* = 40 *cm*^−1^, as shown in Fig. 3(b), although, the distinguishing features of peaks and dips are still present, the absolute values of the response function are much smaller than at the energy of the small gap. In addition, sign change also occurs in the response function for some momenta in the case for *s*_++_ symmetry. The response function starts to lose its indicative character for symmetry of the superconductivity. At energies close to Δ_*a*_, as shown in Fig. 3(c), the behaviour of QPI response function is indistinguishable (apart from the intensity of the response function) in any qualitative manner and hence gives support to our assertion that, only the region of Δ_*b*_ is useful for probing the response behaviour in order to ascertain the nature of gap symmetry in an unambiguous way.

**Figure 4:**
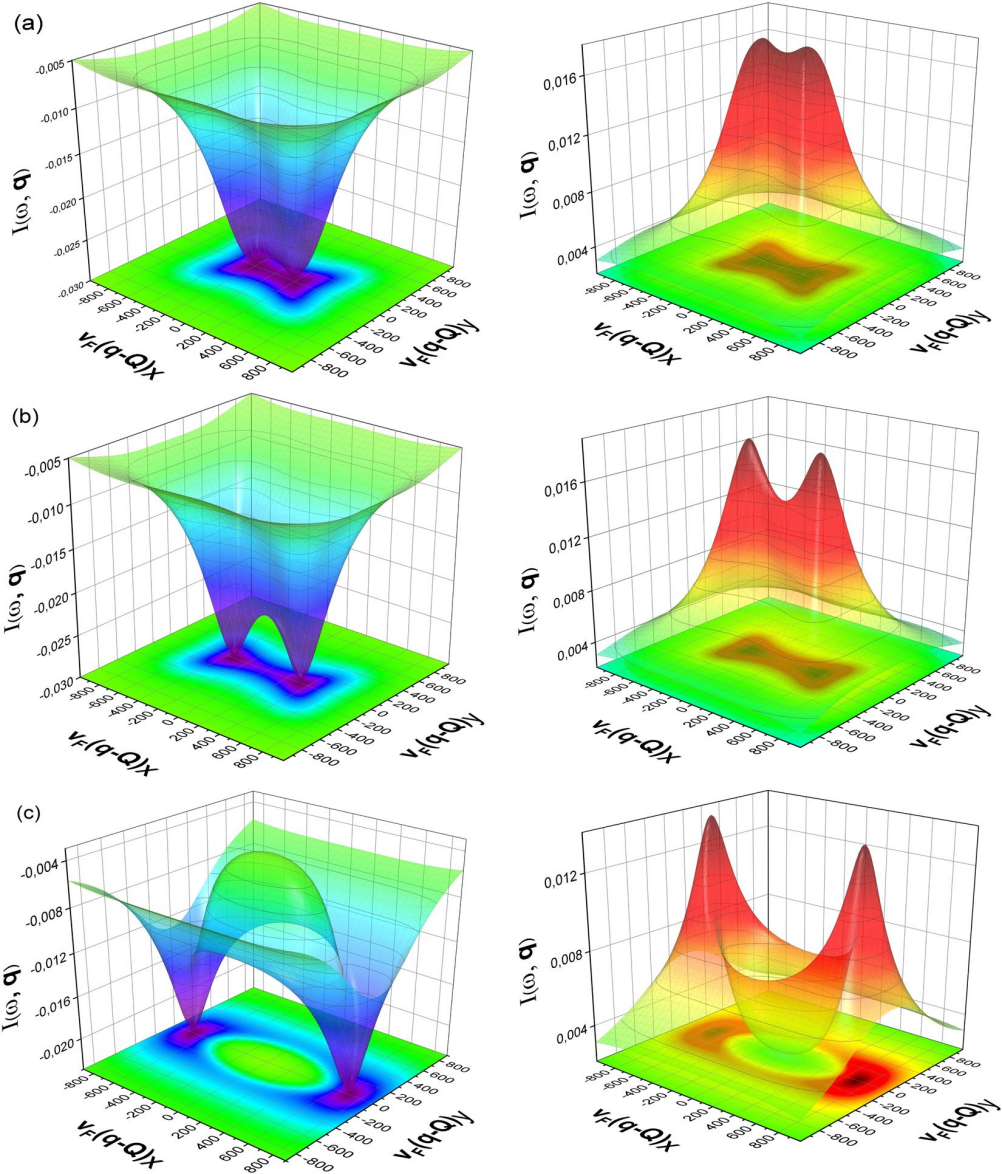
Evolution of momenta dependence of the QPI response function with Fermi-energy mismatch *δμ* = (**a**) 100, (**b**) 200 and (**c**) 500; with *ε* = 200 and *β* = −1 at *ω* = Δ_*b*_ = 18. Here, the panels (**a**),(**b**),(**c**) correspond to the *s*_±_ (left) and *s*_++_ (right), respectively.

In Fig. 4 we present the effect of finite ellipticity on the response function in the presence of various chemical potentials. We choose three categories for Fermi surface mismatch i.e. $\delta \mu  < \varepsilon$, *δμ* = *ε* and $\delta \mu  > \varepsilon$ at fixed electron band ellipticity *ε* = 200. In Fig. 4(a) we observe a diffused dip/peak across the ${{\bf{v}}}_{F}\tilde{{\bf{q}}}$ values for *s*_++_ and *s*_±_. When the value of *δμ* becomes equal to the ellipticity, as in Fig. 4(b), we can clearly observe the shifting of dips/peaks towards larger momenta along ${{\bf{v}}}_{F}{\tilde{{\bf{q}}}}_{x}$ axis and also their sharpening. Moreover, at low ${{\bf{v}}}_{F}\tilde{{\bf{q}}}$ values the appearance of a peak/dip in the *s*_±_ and *s*_++_ case is a new feature. This becomes much prominent for the case $\delta \mu  > \varepsilon$, as in Fig. 4(c), where it covers a large region of momentum value.

Hence, for large Fermi surface mismatch, at a given band ellipticity, the response peaks are located at higher ${{\bf{v}}}_{F}\tilde{{\bf{q}}}$ values. The distinction between *s*_++_ and *s*_±_ symmetry cases is still a robust feature that helps to determine the nature of pairing symmetry.

### The case of the Fermi-pockets with the same nature (e-e or h-h)

**Figure 5:**
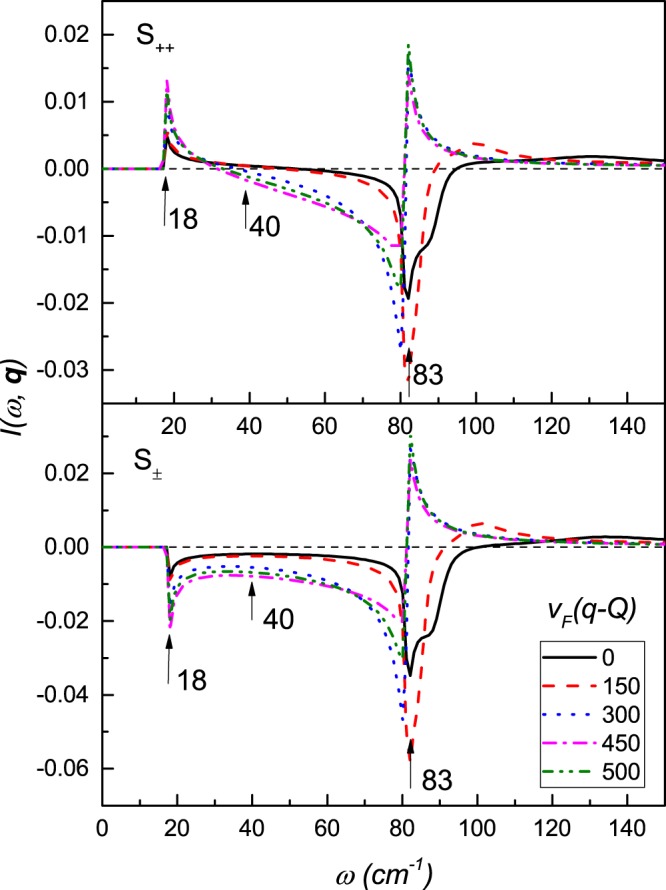
The QPI response function I(*ω*, **q**) of the scattering between the electron and the hole bands, for the *s*_++_ and *s*_±_ symmetry of the superconducting order parameter at various momentum values with parameters as, *ε* = 200, *δμ* = 300, and *ϕ* = 0 with *β* = 1, and at temperature *T* = 1 *cm*^−1^.

In the light of recent theoretical results obtained^39–43^ with only electron pockets, we study and present the variation in response function behaviour, when sgn(*β*) is +1, with the 2D plot given in Fig. 5. We observe that the response peak at Δ_*b*_ depends on the symmetry of the superconducting order parameter, while, at the energy of the large gap Δ_*a*_ the response function is the same for *s*_++_ and *s*_±_ symmetry. In general, the behaviour of the response function for the scattering between two electron bands, and the two hole bands is very similar to those described above for the scattering between electron and hole bands.

**Figure 6:**
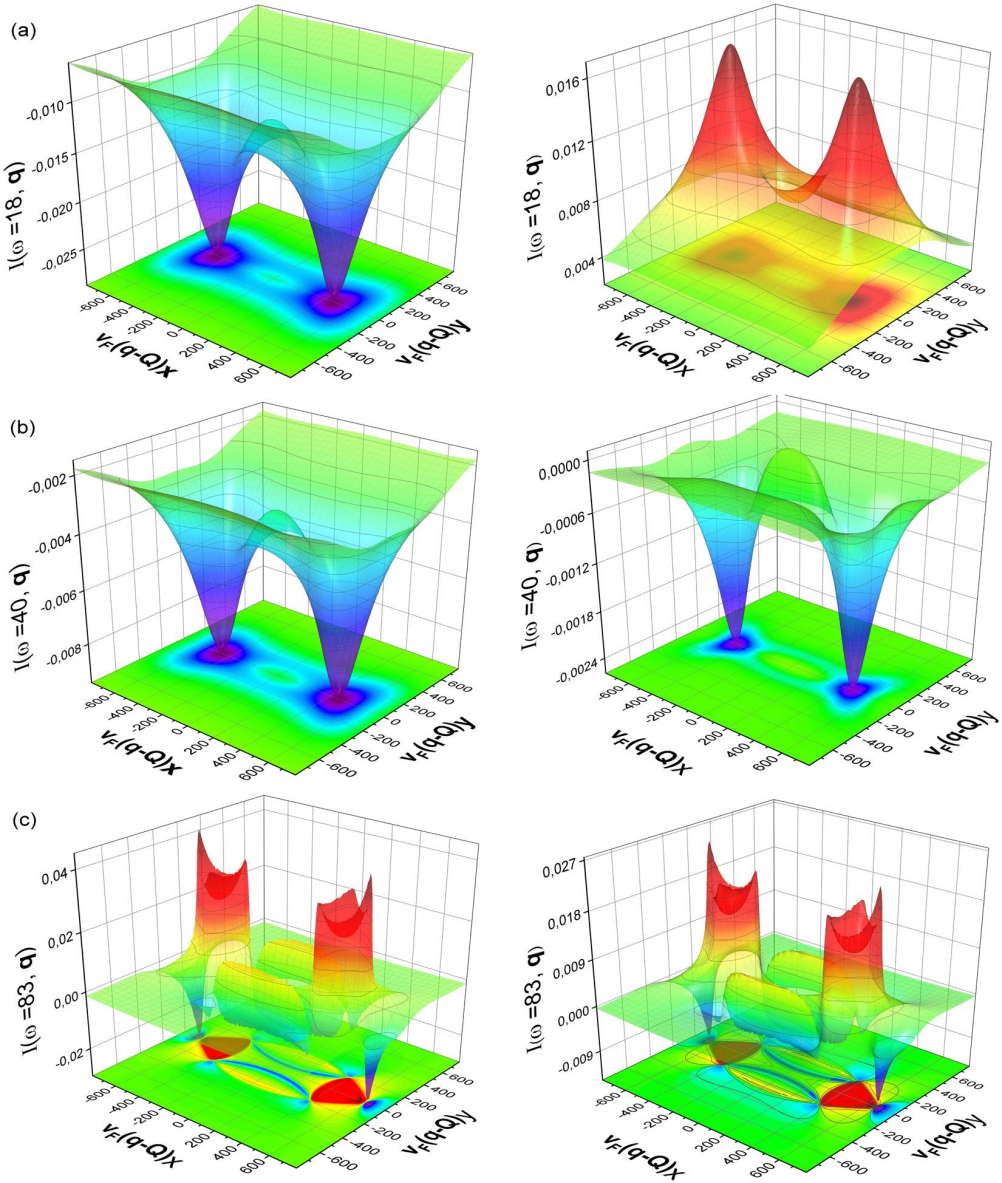
The momentum dependence of the QPI response function with parameters as *δμ* = 300 and *ε* = 200 at certain energies, for *β* = 1. Here, the panels (**a**),(**b**),(**c**) correspond to the *s*_±_ (left) and *s*_++_ (right), respectively.

The mentioned above similarity to the scattering between an electron and a hole bands one can be observed in momenta dependencies of the response function shown in Fig. 6. The comparison of the Figs 3 and 6 shows that the momenta dependence is similar for *ω* = Δ_*b*_. A small difference for other energies are not universal and may disappear with the variation of energy. It confirms the conclusion of^18^ that the QPI at the smaller band gap is a universal feature and may be used as an indicator of the symmetry of the superconducting order parameter.

## Conclusion

We have analyzed the momentum and energy dependence of the QPI response function in the multiband superconductors in the presence of various band parameters. Within an effective two band model it was shown that the more informative is a behavior of this function near the at energies close to the small gap. It has been demonstrated that these dependencies can be used for identification of the superconducting order parameter in FeBS. We have found some peculiarities in the momentum dependence, which are related to the geometry of the electron and hole bands. These features may be important for identifying the Fermi pockets in the experiment. We have shown that the QPI response functions for *s*_±_ and *s*_++_ order parameters are very similar for the energies at the largest gap functions and higher, but different at the energy of the smallest gap function. The result lends support to the assertion that QPI is indeed a useful phase-sensitive technique^18^, within a certain energy range and, hence, may help to determine pairing symmetry and the nature of superconducting order parameter in Fe-based superconductors.
